# Spatially constrained ICA from a language task enables sensorimotor network extraction in patients with space-occupying brain lesions

**DOI:** 10.1007/s11682-026-01183-1

**Published:** 2026-08-12

**Authors:** Eva Calderón-Rubio, Víctor Costumero, Vicente Belloch, César Ávila

**Affiliations:** 1 Neuropsychology & Functional Neuroimaging Group, Universitat Jaume I, Vicent Sos Banyat Avenue Research Building II, Castelló de la Plana (Castellón), 12006 Spain; 2 Department of Radiology, Clinica Ascires, Valencia, Valencia Spain

**Keywords:** fMRI, Sensorimotor cortex, ICA, Space-occupying lesion

## Abstract

Although resting-state fMRI is a promising alternative to task-fMRI in presurgical mapping, protocols to simultaneously assess language lateralization and sensorimotor networks within a single acquisition remain limited. To evaluate the feasibility of using connectivity analysis derived from a language fMRI task to assess language lateralization and extract the sensorimotor network in presurgical patients with space-occupying lesions. In a retrospective study, 40 presurgical patients underwent a verb generation task (VGT) and a hand motor task (HMT). Single-subject spatially-constrained ICA (scICA) was performed on VGT scans to extract sensorimotor components. Sensitivity, specificity and Dice coefficient between scICA-derived scans and HMT activation maps were calculated. The effects of different variables were analyzed using ANOVA. Forty patients (mean age, 40.50 ± 13.99; 21 men) were included. Sensorimotor components could be extracted in all patients within the predefined constrained scICA framework. Using HMT activation as comparative reference, mean ipsilesional voxelwise sensitivity, specificity and Dice coefficient of scICA-derived maps were 78%, 75% and 36%, respectively. No significant differences were found between ipsilesional and contralesional hemispheres in sensitivity or specificity. Exploratory analyses suggested higher sensitivity values in the right contralesional hemisphere, higher magnetic field strength, and typical language lateralization, although most effects did not survive FDR correction. Our study supports the feasibility of extracting sensorimotor-related networks from a language task while simultaneously determining language lateralization in patients with space-occupying lesions. This approach may provide complementary presurgical information, potentially reducing acquisition time. Further validation using direct cortical stimulation and larger prospective cohorts is warranted.

**Supplementary Information:**

The online version contains supplementary material available at https://doi.org/10.1007/s11682-026-01183-1.

## Introduction

Brain lesion surgery requires maximizing resection while preserving cognitive and sensorimotor functions (Lakhani et al., 2023). Although task-based fMRI (TB) is widely used for presurgical motor and language mapping (Buchbinder, 2016), it depends on patient cooperation and performance, limiting its use in cognitively impaired or poorly compliant patients (Leuthardt et al., 2015; Pujol et al., 1998).

Resting-state (RS) fMRI has emerged as a promising alternative to overcome task-based fMRI limitations, as it does not require active patient participation and enables assessment of multiple functional networks (Parker Jones et al., 2017; Seitzman et al., 2019; Sparacia et al., 2021). Although RS has shown promising results for motor mapping (Qiu et al., 2014), language mapping remains less reliable, particularly in atypical lateralization cases (Branco et al., 2016; Lu et al., 2017; Ott et al., 2021; Rolinski et al., 2020; Sair et al., 2017; Sparacia et al., 2019; Tie et al., 2014).

Given the difficulty of using RS to determine language lateralization, an alternative is to use a single language task and apply ICA to identify sensorimotor network (SMN) (Beheshtian et al., 2021; Jurkiewicz et al., 2018). However, limitations remain: (Jurkiewicz et al., 2018) performs a seed-based analysis of normalized data in a healthy subject sample that can be limiting when working in native space of presurgical patients diagnosed with space-occupying lesions; and (Beheshtian et al., 2021) did not detect SMN independent components (IC) in all participants and reported RS–TB correspondence in only 75% of cases.

Our objective in this study is to overcome the above-mentioned limitations, and to do so we propose a novel methodology that uses a language task-based paradigm to pursue three goals: (1) identify language lateralization; (2) extract SMN-related components; and (3) assess the sensitivity, specificity and Dice coefficient (DC) of these results in comparison with a motor fMRI task (Qiu et al., 2014, 2017; Rosazza et al., 2014). To address the shortcomings of previous approaches, we apply spatially constrained ICA (scICA) to language-task preprocessed scans. scICA automatically extracts the ICs of interest using a predefined spatial template that serves as a constraint on the source matrix (Lin et al., 2010; Lu & Rajapakse, 2005). Our main hypothesis is that a single language task may provide information about language lateralization and SMN through scICA.

## Materials and methods

### Participants

A group of 40 patients (21 males, ages 17–78 years, 40.50 ± 13.99) was selected retrospectively (2009–2022) from a pool of 288 cases for meeting the following inclusion criteria: (1) a space-occupying brain lesion candidate for neurosurgery; (2) a motor task for hand movement (HMT); and (3) a verb-generation task (VGT). Exclusion criteria were incomplete tasks, motion artifacts, or poor in-scanner performance.

Demographic data, pathology information, and other variables of interest are reported in Table 1. Prior to scanning, all patients signed an informed consent form, providing written consent to participate in the study. The study protocol was approved by the university Ethics Committee.

**Table 1:** Demographic data, lesion location, pathologic anatomy analysis results, language lateralization (LI), and type of scanner

Parameters	Value
**Midline shift**	13
**Ipsilesional central sulcus displacement**	8
**Lesion location**	
*Hemisphere: left / right*	29 / 11
*Frontal lesion*	30
*Parietal lesion*	2
*Temporoparietal lesion*	2
*Frontotemporal lesion*	2
*Frontoparietal lesion*	4
**Anatomic pathology result**	
*Oligodendroglioma*	5
*Glioma*	5
*IDH-wildtype glioblastoma*	3
*Anaplastic astrocytoma*	2
*Diffuse astrocytoma*	4
*Oligoastrocytoma*	1
*Metastatic lesion*	2
*Meningioma*	2
*Cavernoma*	2
*Cavernous hemangioma*	1
*Hemangiopericytoma*	1
*DNET*	2
*Undetermined space-occupying brain lesion*	10
**Scanner**	
*Siemens TrioTim 3T*	14
*Siemens Sonata 1.5T*	14
*Philips Achieva 3T*	12

Note. – Values are number of patients. “Undetermined space-occupying brain lesion” refers to cases in which definitive histopathological information was unavailable at retrospective review

### Image Acquisition

Three different scanners were used: Siemens Magnetom Trio 3T, Siemens Magnetom Sonata 1.5T and Philips Achieva 3T X-series. First, a 3D structural MRI was acquired using a T1-weighted magnetization-prepared rapid acquisition gradient-echo sequence. Then, a gradient-echo T2*-weighted echo-planar imaging sequence was performed for VGT and HMT. Scan parameters are described in supplementary materials.

### fMRI paradigms

Two fMRI tasks were used: VGT and HMT. For the VGT, 12 blocks of alternating control and activation conditions were performed. After a 6-s fixation period, participants completed the 6-min task composed of 30-s blocks. During activation, participants had to generate single verbs for concrete nouns presented visually every three seconds (ten nouns per block). During the control condition, participants were required to silently repeat two letters presented among four symbols. Participants practiced the task overtly before scanning but performed it silently during acquisition to reduce speech-related motion artifacts (Sanjuán et al., 2010). In HMT, adapted from Pujol et al., 1998, participants repeatedly flexed and extended the fingers of each hand at approximately one cycle per second. Over six minutes, they alternated right- and left-hand movements in 30-second blocks without intermediate rest periods. Patients always began with the hand ipsilateral to the cerebral lesion, which served as the control condition, whereas movement of the contralateral hand served as the activation condition (e.g., left hemisphere lesion: left hand control, right hand activation). The instruction to switch hands was delivered visually on the screen. Participants practiced the task before scanning and were instructed to maintain a constant rhythm while avoiding arm or head movements.

### fMRI task analysis

TB data sets were processed by the SPM12 software package (Wellcome Trust Centre for Neuroimaging, London, UK) in native space.

HMT pre-processing included: (1) alignment of images to AC-PC plane; (2) head motion correction, realignment and reslicing; and (3) spatial smoothing (FWHM=4 mm). The GLM was applied by defining a boxcar function representing activation and control blocks convolved with the hemodynamic response function. Realignment parameters were added as nuisance variables and a high-pass (128s) filter was applied. Finally, individual-level one-sample t-tests were conducted at *p*=.0001 (uncorrected) and cluster-level family-wise error (FWE) corrected at *p*<.05 (standard threshold routinely used by our group for presurgical motor mapping), to obtain functional SMN maps for voxelwise performance analyses.

Pre-processing for the VGT data followed the same steps as previously described, with the addition of the following procedures between steps 2 and 3: co-registration of the T1-weighted structural images to the mean functional image; and segmentation of T1 structural images, applying an inverse deformation field. A normalized pipeline was used to determine group activation in both language and motor tasks and to calculate language lateralization index (see Supplementary Materials).

### scICA

The Neuromark 2.1 template including 105 non-artifactual networks was used as a spatial constraint (https://trendscenter.org/data/) (Iraji et al., 2022). The SMN spanned IC62 to IC74; however, only IC72 and IC73 fully encompassed the right and left central sulcus, respectively—from superior to inferior—including the characteristic omega-shaped hand knob. Therefore, these two components were extracted to represent the right and left SMN. Inverse deformation fields of VGT obtained from the segmentation step were applied to these templates for each patient. The resulting warped templates were spatially smoothed (FWHM=4 mm).

Single-subject scICA was performed separately for each patient on VGT fMRI smoothed scans in native space by means of Group ICA of fMRI Toolbox (GIFT v1.3 h, Medical Image Analysis Lab, http://mialab.mrn.org/software/gift). Number of IC was set to 2, as these components corresponded to the bilateral dorsal SMN relevant to the study, and “Constrained ICA (Spatial)” was selected as the analysis algorithm. Then, all remaining parameters were left at their default values.

IC72 and IC73 maps, thresholded at z = 1.000 (one standard deviation above the mean), were combined for each patient and the resulting image was overlapped to each T1 scan. For each participant, a volume of interest was defined using MRIcron (Rorden & Brett, 2000) including central sulcus, precentral gyrus and postcentral gyrus, serving as scICA-derived SMN. We also confirmed that the hand activation maps of all patients were spatially aligned within the previously defined anatomical landmarks. Unlike previous studies (Beheshtian et al., 2021), our method aims to reduce subjectivity by relying on predefined anatomical boundaries rather than manual component selection or inter-rater agreement.

### Statistical analysis

For visualization purposes only, group-level one-sample t-tests were performed for the language task (separating typical and atypical cases) and for the motor task (separating left and right activation) at voxel-wise *p*<.001 (uncorrected) and cluster-level (FWE) corrected at *p*<.05.

Individual HMT maps used for voxelwise sensitivity, specificity and DC analyses were obtained using single-subject one-sample t-tests thresholded at *p*=.0001 (uncorrected), and cluster-level FWE corrected at *p*<.05, corresponding to the standard threshold routinely used by our group for presurgical motor mapping. Performance metrics (sensitivity, specificity and DC) were derived from these individual maps.

Number of voxels of functional (HMT) and scICA-derived SMN (IC72/IC73) and their overlap was calculated, taking active voxels in the HMT as the comparative reference. True positive, true negative, false positive and false negative values were obtained for each patient. To determine true negative values, subject-specific anatomical masks derived from the original IC72 and IC73 Neuromark templates were transformed to each patient’s native space before GIFT processing and used as a reference landmark. Voxels not classified as positive either in the HMT activation map or in the scICA-derived SMN were considered true negatives. Then, sensitivity (true positives/true positives + false negatives) and specificity (true negatives/true negatives + false positives) were calculated. Number of voxels was determined as the standard unit of measure for both methods and to conduct the sensitivity and specificity analysis. A pragmatic 50% overlap reference value was additionally considered for sensitivity interpretation. Predictions meeting or exceeding this threshold were classified as successfully localized. Next, DC was calculated following the formula DC=2xA∩B/(A + B), where A is the number of voxels of HMT, B the number of voxels of SMN (IC72/73) and A∩B is the overlap (Dice, 1945).

One factor exploratory ANOVA was performed to analyze differences between sensitivity, specificity and DC values across sample variables. ANOVA results were corrected for multiple comparisons using the Benjamini-Hochberg False Discovery Rate (FDR) with *q*<0.05. For completeness, uncorrected p-values are also shown to illustrate subthreshold trends. All statistical analyses were conducted using SPSS (IBM SPSS Statistics, v.28).

## Results

**Fig. 1:**
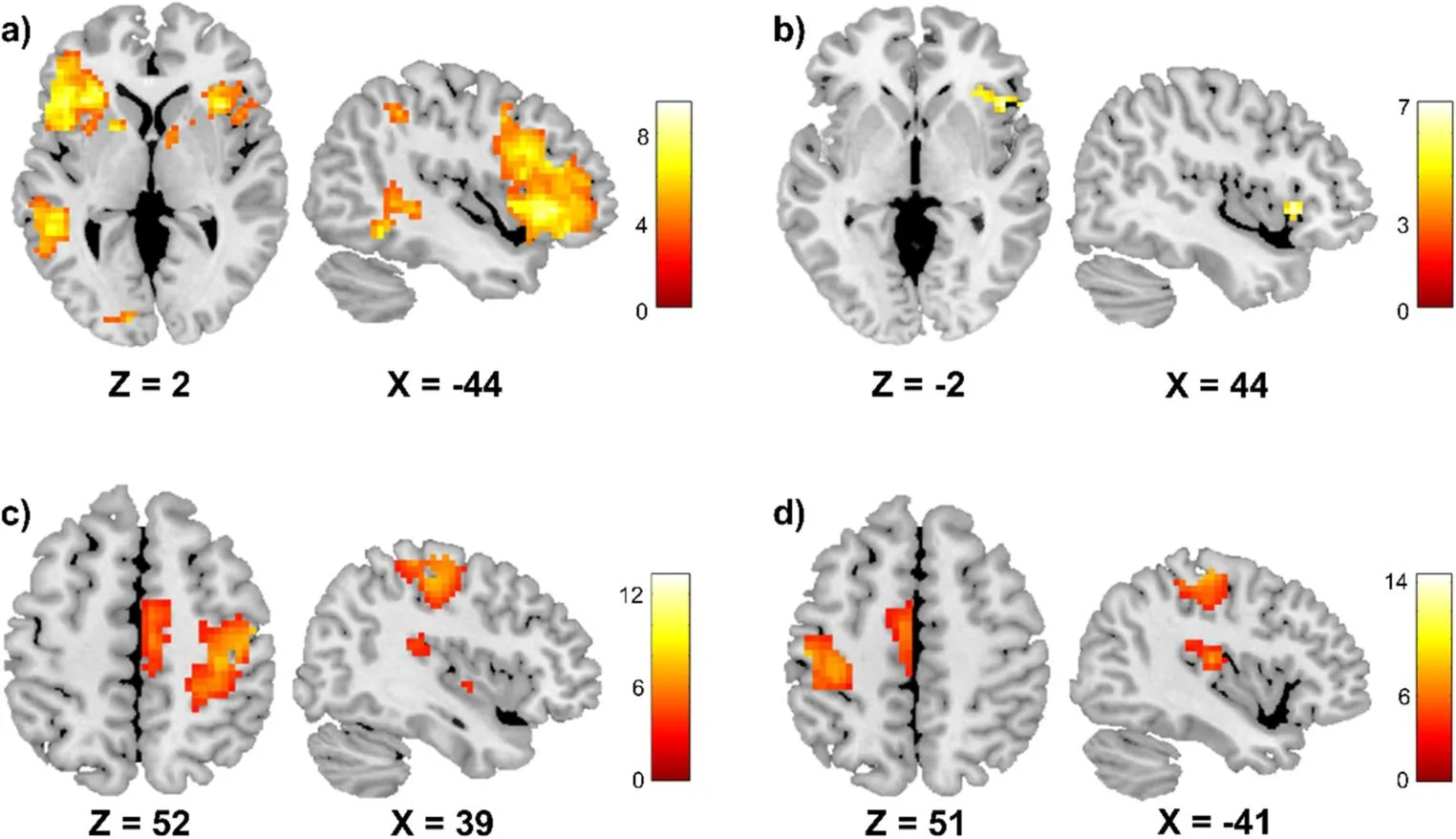
Descriptive group-level t-maps of **a**) verb generation task in patients with a typical lateralization of language (N=29), **b**) verb generation task in patients with an atypical lateralization of language (N=11), **c**) hand motor task in right hemisphere (ipsilesional hemisphere: N=11; contralesional hemisphere: N=29), and **d**) hand motor task in left hemisphere (ipsilesional hemisphere: N=29; contralesional hemisphere: N=11) in MNI space (see supplementary text 1). Voxel-wise threshold at *p*< .001, FWE cluster-corrected at *p*< .05. Coordinates reported in MNI space. Color bars represent t values

VGT showed group activation in the inferior frontal gyrus (IFG), including pars opercularis, and triangularis but also posterior areas (Fig. 1a and b). Group-level activations for descriptive task visualization were observed at voxel-wise *p*<.001 (uncorrected) and cluster-level FWE corrected at *p*<.05 in 38 patients in the left IFG, in 22 patients in right IFG, in 34 patients in the left temporal cortex and in 10 patients the right temporal cortex. LIs calculated in the IFG showed a mean of 45.65 (SD = 36.54) in the full sample. Typical left lateralization was observed for 29 patients (M = 64.07; SD = 9.415; range: 43–76), while 11 patients exhibited atypical language lateralization (M=-2.91; SD = 37.163; range: -59–39). This atypical lateralization was observed in one patient with left parietal lesion, nine patients with left frontal lesions, and one patient with left frontoparietal lesion.

The HMT task revealed reliable group activations in the ipsilesional primary motor cortex, sensorimotor cortex and supplementary motor area (voxel-wise *p*<.001 (uncorrected) and cluster-level FWE corrected at *p*<.05; Fig. 1c and d). Our procedure also obtained significant activations of contralesional motor area in all patients when the opposite contrast (control> activation) was applied. Eight patients (8/40, 20%) exhibited displacement of the central sulcus involving the SMN. In these patients, the displaced omega-shaped hand knob remained functionally active, suggesting preservation of functional activation, without excluding subtle or distributed reorganization. 

### ICA analysis

**Fig. 2:**
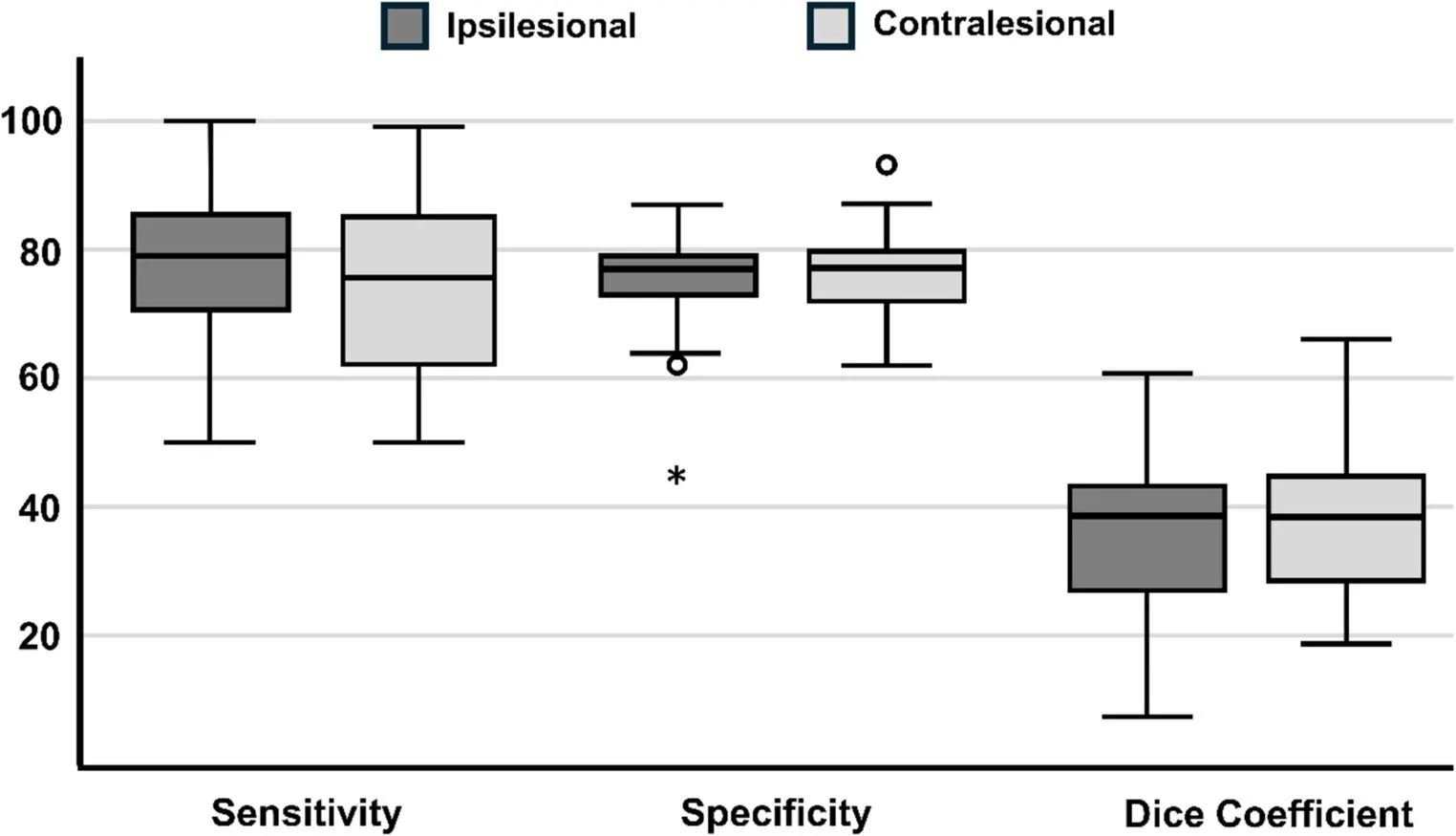
Boxplot of the distribution of sensitivity, specificity and Dice coefficient values for ipsilesional and contralesional sensorimotor network maps identified within the predefined constrained scICA framework. Boxes represent the interquartile range and median; whiskers indicate the range of values. Values on the Y-axis are expressed as percentages. Circles indicate outliers and the asterisk an extreme outlier

The scICA prededefined constrained framework enabled the extraction of SMN components in all 40 patients, with sensitivity values ≥ 50% in all cases. To determine the validity of this SMN estimation, we compared the activated voxels with those obtained with the native HMT (*p*=.0001). (Fig. 2) summarizes the distribution of sensitivity, specificity and DC values for both hemispheres, illustrating inter-subject variability in method performance. Ranges were 50%-100%, 44.81%-87.07%, and 7.66%-59.78% for the ipsilesional hemisphere, and 50%-99.48%, 61.48%-92.65%, and 18.64%-59.15% for the contralesional hemisphere, respectively. 

To provide a more comprehensive description of classification performance, additional confusion-matrix metrics, including number of voxels of predicted and functional SMN, true positives, true negatives, false positives, false negatives, sensitivity and specificity, are reported for each hemisphere and each patient in the supplementary materials. These metrics allow a patient-level evaluation of scICA-based SMN-derived connectivity relative to the HMT comparative reference. Finally, paired-sample t-tests showed no significant differences between ipsilesional and contralesional sensitivity (*t*(39) = 1.68, *p*=.10, d=0.17), specificity (*t*(39) = − 1.62, *p*=.11, d=0.06) or DC (t(39)=0.92, *p*=.37, d=0.10), indicating comparable performance of the method regardless of lesion side.

In the 8 patients with displacement of the central sulcus, the mean sensitivity, specificity and DC of the SMN map extracted from scICA and compared to HMT were 80% (SD = 14%, 95%CI [69–92]), 70% (SD = 13%, 95%CI [59–80]) and 29% (SD = 9%, 95%CI [18–39]) in the ipsilateral hemisphere, and 77% (SD = 13%, 95%CI [66–87]), 79% (SD = 9%, 95%CI [71–86]) and 34% (SD = 10%, 95%CI [26–42]), in the contralateral hemisphere, respectively.

Different exploratory ANOVAs were run to investigate the role of different acquisition and clinical variables (Table 2 for results). These suggested that 3T scanners produced higher ipsilesional and contralesional sensitivity and ipsilesional DC than 1.5T scanners. Also, contralesional sensitivity appeared higher in the right hemisphere than in the left, and for typical than atypical lateralization of language. Finally, ipsilesional specificity and DC appeared higher when the central sulcus was not displaced by the space-occupying lesion. After applying Benjamini–Hochberg FDR correction to the 10 univariate ANOVAs per metric, only the effect of language lateralization on contralesional sensitivity remained significant (*p*_FDR=0.010). No other comparisons survived FDR correction.

**Table 2:** F-values obtained in ANOVAs for variables type of lesion, lesion localization, lesion lobe, hemisphere, scanner, scanner (1.5T vs. 3T), language lateralization, midline shift and ipsilesional central sulcus displacement

Variables	Sensitivity	Specificity	Dice coefficient
**Ipsilesional**			
*Hemisphere (left vs. right)*	1.27 (0.27), ηρ^2^ = 0.03	0.87 (0.36), ηρ^2^ = 0.02	0.62 (0.44), ηρ^2^ = 0.01
*Scanner (1.5T vs. 3T)*	5.13 (0.03), ηρ^2^ = 0.12**	0.23 (0.64), ηρ^2^ = 0.01	7.62 (0.009), ηρ^2^ = 0.17**
*Language lateralization (typical vs. atypical)*	0.16 (0.69), ηρ^2^ = 0.00	1.98 (0.17), ηρ^2^ = 0.05	1.21 (0.28), ηρ^2^ = 0.03
*Midline shift (yes vs. no)*	0.007 (0.93), ηρ^2^ = 0.00	0.04 (0.85), ηρ^2^ = 0.00	0.500 (0.48), ηρ^2^ = 0.01
*Central sulcus displacement (yes vs. no)*	0.31 (0.58), ηρ^2^ = 0.01	6.47 (0.02), ηρ^2^ = 0.15**	4.56 (0.04), ηρ^2^ = 0.11**
**Contralesional**			
*Hemisphere*	4.58 (0.04), ηρ^2^ = 0.10**	2.13 (0.15), ηρ^2^ = 0.05	0.44 (0.51), ηρ^2^ = 0.01
*Scanner (1.5T vs. 3T)*	4.17 (0.05), ηρ^2^ = 0.10**	3.68 (0.06), ηρ^2^ = 0.09	3.81 (0.06), ηρ^2^ = 0.09
*Language lateralization (typical vs. atypical)*	16.26 (< 0.001), ηρ^2^ = 0.30*	1.66 (0.21), ηρ^2^ = 0.04	2.31 (0.14), ηρ^2^ = 0.06
*Midline shift (yes vs. no)*	2.21 (0.15), ηρ^2^ = 0.06	0.46 (0.50), ηρ^2^ = 0.01	1.29 (0.26), ηρ^2^ = 0.03
*Central sulcus displacement (yes vs. no)*	0.47 (0.500), ηρ^2^ = 0.01	1.03 (0.32), ηρ^2^ = 0.03	0.78 (0.38), ηρ^2^ = 0.02

Note. – Data are presented as Fisher’s F (p value), with partial eta squared (ηp²) reported as effect size. P values shown in the table are uncorrected. Significant results surviving FDR correction (q < .05) are marked with *. Nominally significant uncorrected effects (p < .05) that did not survive FDR correction are marked with **

**Fig. 3:**
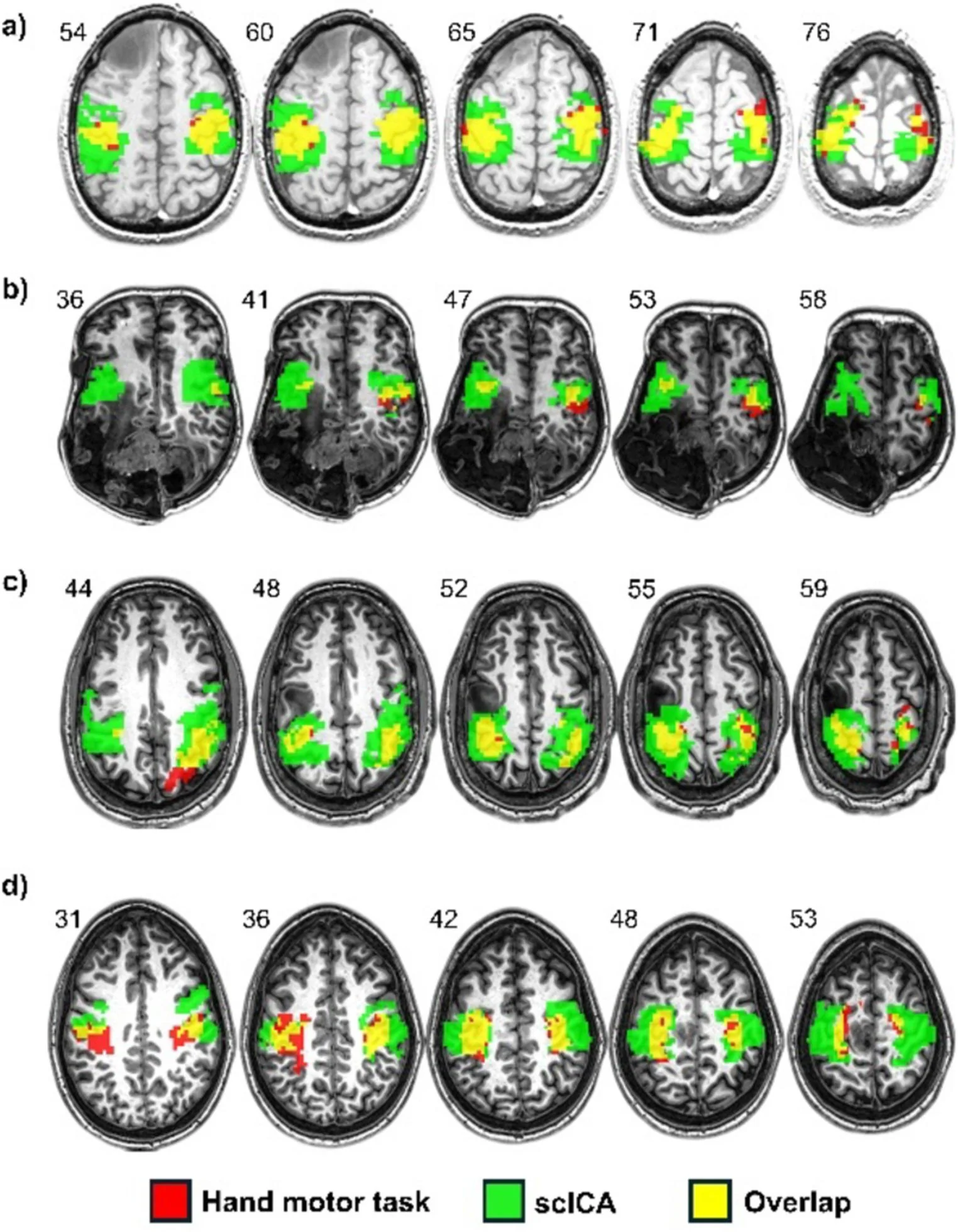
Overlap of hand motor task and SMN components extracted from scICA framework. Green overlay: sensorimotor network obtained from volume of interest of the combination of IC72 and IC73 of each patient’s scICA; red overlays: hand functional activation extracted from each patient’s hand motor task in native space; yellow overlays: overlapping regions. **a**) 32-year-old man with left frontal diffuse astrocytoma. Sensitivity, specificity and Dice coefficient (%) for the ipsilesional hemisphere were 90.00, 79.99 and 51.69, respectively; for the contralesional hemisphere, 86.47, 81.87 and 59.15, respectively. **b**) 23-year-old woman with frontoparietal hemangiopericytoma. Sensitivity, specificity and Dice coefficient (%) for the ipsilesional hemisphere were 100.00, 79.81 and 7.66, respectively; for the contralesional hemisphere, 62.02, 84.97 and 20.18, respectively. **c**) 33-year-old man with left frontal diffuse astrocytoma. Sensitivity, specificity and Dice coefficient (%) for the ipsilesional hemisphere were 95.08, 77.50 and 38.89, respectively; for the contralesional hemisphere, 80.65, 77.94 and 39.85, respectively. **d**) 23-year-old woman with left frontal oligodendroglioma. Sensitivity, specificity and Dice coefficient (%) for the ipsilesional hemisphere were 89.72, 79.95 and 59.22, respectively; for the contralesional hemisphere, 94.80, 77.92 and 47.36, respectively. scICA=spatially constrained independent component analysis

To illustrate individual results, (Fig. 3) shows hand activation obtained from HMT at voxel-wise *p*=.0001 (uncorrected) and cluster-level FWE corrected at *p*<.05 overlapping scICA-derived SMN in four sample patients. A 3D video of results displayed in (Fig. 3) can be seen in supplementary materials. 

## Discussion

In this study, we introduce a feasibility framework for extracting SMN components from a single 6-minute language protocol in presurgical patients with space-occupying lesions. The VGT paradigm provided language lateralization-related activation patterns, particularly within the IFG, consistent with previous literature in both healthy participants and clinical populations (Sanjuán et al., 2010; Villar-Rodríguez et al., 2020). Applying scICA to the language scans, sensorimotor-related components were extracted in all patients, including those exhibiting evident displacement of the central sulcus due to lesion-related mass effect (Beheshtian et al., 2021). Dice coefficient values indicated modest-to-moderate overlap, which is expected given the broader spatial extent of connectivity-derived networks relative to task-based activation maps. In summary, these findings support the potential utility of constrained ICA approaches for obtaining complementary motor-related information from a single language-task acquisition.

Few previous studies have used language tasks to estimate SMN, and none of them employ quantitative measures to analyze the overlap between methods (Beheshtian et al., 2021; Jurkiewicz et al., 2018; Sparacia et al., 2019). Our sensitivity and specificity values were calculated at the voxel level by comparing the overlap between the SMN network obtained through scICA and the activation derived from the HMT. Using this protocol, voxel-wise sensitivity was good (78% ipsilesional; 74% contralesional), with 80% of cases showing good–excellent performance. Ipsilesional specificity averaged 75%, indicating a relatively low false-positives rate, with a small number of outlying values observed across subjects. These results were comparable even in the presence of mass effect, which displaced the sensorimotor areas. This opens the possibility of using structural T1-weighted images with anatomical masks and inverse deformations for rapid functional mapping. Future studies should explore this possibility.

When comparing sensitivity with previous studies, one work did not report voxelwise sensitivity (Beheshtian et al., 2021), while another found lower sensitivity values (44%, Rosazza et al., 2014) when comparing RS and TB using ROIs or ICA. In contrast, using direct cortical stimulation as reference, both lower (63%; Qiu et al., 2017) and higher values (78–90%; Qiu et al., 2014) have been reported. However, those comparisons rely on non-equivalent units, and larger surface-based regions can inflate sensitivity estimates. Thus, our voxel-wise approach applies a stricter criterion, making results more comparable across studies.

Although two previous works have reported higher specificity values (84–89%, Qiu et al., 2014; 93%, Qiu et al., 2017) when localizing motor areas, these results were obtained using direct cortical stimulation as the gold standard. While this technique is considered highly accurate, it differs fundamentally from our reference (fMRI functional task). As discussed previously, comparisons across studies must consider the variability introduced by the comparative reference. In contrast, the study most comparable to ours —based on ICA and RS— reported considerably lower specificity values (27–51%, Rosazza et al., 2014). In this context, our mean specificity of 75% in the ipsilesional hemisphere compares favorably with the values reported by Rosazza et al. (2014). However, because specificity was calculated within predefined perirolandic masks related to the scICA framework, it should be interpreted as reflecting performance within a constrained search space rather than an unbiased estimate of false-positive rate. These findings nevertheless support the potential utility of this approach in presurgical research.

Previous studies using Dice coefficient reported spatial overlap values around 28–33% (Sair et al., 2016). In our cohort, mean DC were 36% and 37% for the ipsilesional and contralesional hemispheres, respectively, indicating moderate spatial correspondence between scICA-derived and task-based SMN maps, consistent with previous literature (Niazy et al., 2011). These values should be interpreted considering the broader spatial extent of connectivity-derived networks obtained with scICA compared with the more focal nature of task-based activation maps, which inherently limits voxel-wise overlap.

Exploratory analyses suggested slightly higher performance at 3T than 1.5T, possibly due to improved spatial resolution and signal-to-noise ratio (Tyndall et al., 2017). Sensitivity was also reduced in cases with central sulcus displacement and in the contralesional hemisphere of patients with left hemisphere lesions or atypical language lateralization (Lehéricy et al., 2000; Nitschke et al., 1998). However, most effects did not remain significant after correction for multiple comparisons and should therefore be considered exploratory.

### Limitations

Several limitations should be acknowledged. First, thresholds for task-based fMRI and scICA analyses were selected based on previous experience, and different thresholding strategies may influence spatial overlap estimates. Future studies should evaluate robustness across multiple thresholds. Second, the specificity analysis partly relied on subject-specific masks derived from the constrained ICA framework, potentially introducing partial circularity into true negative estimation. In addition, the high extraction rate should be interpreted in the context of the strong anatomical and template priors incorporated into the pipeline, including predefined NeuroMark sensorimotor components and anatomically constrained perirolandic evaluation. Thus, the reported performance reflects guided network extraction rather than fully independent network discovery. Third, the constrained ICA approach used in this study reduces dependence on model-order selection by incorporating spatial priors. Nevertheless, the resulting component maps may still be influenced by the number and set of priors included in the analysis, which should be considered in future applications. Fourth, HMT fMRI was used as a comparative reference rather than a definitive gold standard for motor localization. Direct cortical stimulation would provide stronger validation. Because the HMT used contralateral hand movement as an active control condition, motor maps reflected relative hemispheric dominance patterns, potentially influencing activation extent. Fifth, the study included heterogeneous scanners, lesion characteristics, and language lateralization profiles within a relatively modest sample, so some variables may be partially confounded. Consequently, statistical analysis should be considered as exploratory rather than confirmatory. Furthermore, because inclusion required successful completion of both motor paradigms, the findings primarily apply to task-compliant patients and may not generalize to individuals unable to perform standard task-based fMRI. Finally, only hand motor task data were available; future multicenter studies incorporating mouth and foot paradigms would provide a more comprehensive evaluation.

### Conclusion

In conclusion, this feasibility study demonstrates that spatially constrained ICA applied to language-task fMRI can extract sensorimotor-related networks while simultaneously providing language lateralization information in patients with space-occupying lesions. The observed spatial correspondence between scICA-derived maps and HMT activation supports the potential utility of this approach for obtaining complementary presurgical functional information from a single acquisition. However, further validation using direct cortical stimulation, different thresholds, and larger prospective cohorts is required before clinical implementation.

## Supplementary Information

Below is the link to the electronic supplementary material.

Supplementary Material 1

## Data Availability

All data supporting the findings of this study are available from the corresponding author on reasonable request, in accordance with institutional and ethical regulations.

## Abbreviations

RS: resting-state fMRI
TB: task-based fMRI
SMN: sensorimotor network
DC: Dice coefficient
IC: independent component
scICA: spatially constrained independent component analysis
HMT: hand motor task
VGT: verb generation task
FWE: family-wise error
