# Supplementary material for: Spatially constrained ICA from a language task enables sensorimotor network extraction in patients with space-occupying brain lesions
Source: Brain Imaging Behav. 2026 Aug 12;20(4):120. doi: 10.1007/s11682-026-01183-1 (PMC13469547; doi:10.1007/s11682-026-01183-1)
Supplement: Supplementary file 1 [file 11682_2026_1183_MOESM1_ESM.docx]

**Supplementary table 1. Scan parameters.**

| Scanner | TR/TE (ms) | Matrix | Voxel size | Volumes |
| --- | --- | --- | --- | --- |
| T1-weighted sequence |  |  |  |  |
| Siemens TrioTim 3T | 19/4.92 | 160 x 256 x 256 | 1 x 1 x 1 |  |
| Siemens Sonata 1.5T | 20/4.38 | 160 x 256 x 256 | 1 x 0.98 x 0.98 |  |
| Philips Achieva 3T | 8.15/3.73 | 150 x 256 x 256 | 1 x 1 x 1 |  |
| VGT T2*-weighted sequence |  |  |  |  |
| Siemens TrioTim 3T | 3000/50 | 28 x 64 x 64 | 6 x 4 x 4 | 120 |
| Siemens Sonata 1.5T | 3000/50 | 28 x 64 x 64 | 5 x 3.94 x 3.94 | 120 |
| Philips Achieva 3T | 2500/30 | 31 x 80 x 80 | 4 x 3 x 3 | 144 |
| HMT T2*-weighted sequence |  |  |  |  |
| Siemens TrioTim 3T | 3000/50 | 28 x 64 x 64 | 6 x 4 x 4 | 120 |
| Siemens Sonata 1.5T | 3000/50 | 28 x 64 x 64 | 5 x 3.94 x 3.94 | 120 |
| Philips Achieva 3T | 2000/30 | 31 x 80 x 80 | 4 x 3 x 3 | 120 |

Note. - TR and TE are expressed in milliseconds. Matrix and voxel size data are addressed in millimeters. TR = repetition time, TE = echo time, VGT = verb generation task, HMT = hand motor task.

**Supplementary text 1. Normalized pipeline and language lateralization.**

A normalized pipeline was used to determine group activation in both language and motor tasks: (1) alignment of T1 structural image to AC-PC plane; (2) head motion correction, realigning and reslicing of each fMRI dataset to match the mean functional image; (3) co-registration of T1 structural images to the mean functional image; (4) segmentation of the co-registered images, applying a forward deformation field; (5) spatial normalization of the resliced functional images to the MNI (Montreal Neurological Institute, Montreal, Canada) space with a voxel size of 3x3x3 mm; (6) spatial smooth with 4x4x4 FWHM. The general linear model was applied by defining a T-contrast: *activation > control*. Realignment parameters were added as nuisance variables and a high-pass (128s) filter was applied. Finally, one-sample t-tests were conducted for each task to identify statistically significant active areas (p < .001).

Lateralization index of normalized VGT was calculated for each patient by the LI Toolbox bootstrap method (Wilke & Schmithorst, 2006) (LI-Toolbox for SPM12). The following equation was used to calculate it: [(L – R) / (L + R)] x 100, were L and R are the total amount of significantly active voxels in the left and right hemisphere, respectively. Results range from +100 (totally left-lateralized language) to -100 (totally right-lateralized language). In accordance with previous studies (Villar-Rodríguez et al., 2024), a ± 40 cut-off was used to classify patients in typical (≥ 40) or atypical (right-lateralized: ≤ -40; or ambilateral: between -39 and 39).

Villar-Rodríguez, E., Davydova, T., Marin-Marin, L., & Avila, C. (2024). Atypical lateralization of visuospatial attention can be associated with better or worse performance on line bisection. *Brain Structure and Function*. https://doi.org/10.1007/s00429-024-02822-3

Wilke, M., & Schmithorst, V. J. (2006). A combined bootstrap/histogram analysis approach for computing a lateralization index from neuroimaging data. *NeuroImage*, *33*(2), 522–530. https://doi.org/10.1016/j.neuroimage.2006.07.010

**Supplementary table 2A. Confusion matrix values for hand motor area detection in the ipsilesional hemisphere.**

| ID | A | B | TP | TN | FP | FN |
| --- | --- | --- | --- | --- | --- | --- |
| 1 | 170 | 383 | 138 | 971 | 245 | 32 |
| 2 | 129 | 368 | 98 | 901 | 270 | 31 |
| 3 | 87 | 319 | 79 | 830 | 240 | 8 |
| 4 | 132 | 338 | 108 | 1028 | 230 | 24 |
| 5 | 141 | 502 | 132 | 633 | 370 | 9 |
| 6 | 222 | 351 | 111 | 898 | 240 | 111 |
| 7 | 120 | 414 | 102 | 972 | 312 | 18 |
| 8 | 120 | 275 | 93 | 707 | 182 | 27 |
| 9 | 125 | 335 | 87 | 726 | 248 | 38 |
| 10 | 133 | 430 | 105 | 902 | 325 | 28 |
| 11 | 170 | 422 | 153 | 1075 | 269 | 17 |
| 12 | 262 | 471 | 158 | 731 | 313 | 104 |
| 13 | 49 | 371 | 41 | 873 | 330 | 8 |
| 14 | 134 | 383 | 126 | 695 | 257 | 8 |
| 15 | 211 | 457 | 136 | 800 | 321 | 75 |
| 16 | 93 | 442 | 55 | 1362 | 387 | 38 |
| 17 | 214 | 348 | 127 | 936 | 221 | 87 |
| 18 | 127 | 411 | 77 | 943 | 334 | 50 |
| 19 | 88 | 405 | 62 | 1137 | 343 | 26 |
| 20 | 91 | 462 | 78 | 1162 | 384 | 13 |
| 21 | 129 | 572 | 74 | 930 | 498 | 55 |
| 22 | 120 | 393 | 98 | 1102 | 295 | 22 |
| 23 | 32 | 804 | 32 | 3051 | 772 | 0 |
| 24 | 74 | 287 | 57 | 808 | 230 | 17 |
| 25 | 79 | 333 | 66 | 1063 | 267 | 13 |
| 26 | 87 | 265 | 69 | 1320 | 196 | 18 |
| 27 | 100 | 584 | 85 | 1252 | 499 | 15 |
| 28 | 244 | 949 | 232 | 2469 | 717 | 12 |
| 29 | 78 | 380 | 55 | 893 | 325 | 23 |
| 30 | 259 | 893 | 206 | 2318 | 687 | 53 |
| 31 | 91 | 530 | 62 | 830 | 468 | 29 |
| 32 | 651 | 1042 | 506 | 1898 | 536 | 145 |
| 33 | 230 | 934 | 184 | 2141 | 750 | 46 |
| 34 | 467 | 948 | 419 | 2109 | 529 | 48 |
| 35 | 259 | 1886 | 233 | 1342 | 1653 | 26 |
| 36 | 401 | 983 | 297 | 2596 | 686 | 104 |
| 37 | 407 | 964 | 317 | 2116 | 647 | 90 |
| 38 | 226 | 1080 | 169 | 2059 | 911 | 57 |
| 39 | 403 | 1481 | 307 | 2433 | 1174 | 96 |
| 40 | 180 | 813 | 170 | 1872 | 643 | 10 |
|  |  |  |  |  |  |  |

Note. – Columns 2 to 7 data are number of voxels. Values used to compute ipsilesional sensitivity, and specificity in supplementary table 2B. ID = subject identifier, A = hand motor task voxels, B = scICA voxels of the predicted SMN, TP = true positive (A∩B), TN = true negative, FP = false positive, FN = false negative.

| ID | Sensitivity | Specificity | Dice coefficient |
| --- | --- | --- | --- |
| 1 | 138 / 170 (81.18) | 971 / 1216 (79.85) | 276 / 553 (49.91) |
| 2 | 98 / 129 (75.97) | 901 / 1171 (76.94) | 196 / 497 (39.44) |
| 3 | 79 / 87 (90.80) | 830 / 1070 (77.57) | 158 / 406 (38.92) |
| 4 | 108 / 132 (81.82) | 1028 / 1258 (81.72) | 216 / 470 (45.96) |
| 5 | 132 / 141 (93.62) | 633 / 1003 (63.11) | 264 / 643 (41.06) |
| 6 | 111 / 222 (50.00) | 898 / 1138 (78.91) | 222 / 573 (38.74) |
| 7 | 102 / 120 (85.00) | 972 / 1284 (75.70) | 204 / 534 (38.20) |
| 8 | 93 / 120 (77.50) | 707 / 889 (79.53) | 186 / 395 (47.09) |
| 9 | 87 / 125 (69.60) | 726 / 974 (74.54) | 174 / 460 (37.83) |
| 10 | 105 / 133 (78.95) | 902 / 1227 (73.51) | 210 / 563 (37.30) |
| 11 | 153 / 170 (90.00) | 1075 / 1344 (79.99) | 306 / 592 (51.69) |
| 12 | 158 / 262 (60.31) | 731 / 1044 (70.02) | 316 / 733 (43.11) |
| 13 | 41 / 49 (83.67) | 873 / 1203 (72.57) | 82 / 420 (19.52) |
| 14 | 126 / 134 (94.03) | 695 / 952 (73.00) | 252 / 517 (48.74) |
| 15 | 136 / 211 (64.45) | 800 / 1121 (71.36) | 272 / 668 (40.72) |
| 16 | 55 / 93 (59.14) | 1362 / 1749 (77.87) | 110 / 535 (20.56) |
| 17 | 127 / 214 (59.35) | 936 / 1157 (80.90) | 254 / 562 (45.20) |
| 18 | 77 / 127 (60.63) | 943 / 1277 (73.84) | 154 / 538 (28.62) |
| 19 | 62 / 88 (70.45) | 1137 / 1480 (76.82) | 124 / 493 (25.15) |
| 20 | 78 / 91 (85.71) | 1162 / 1546 (75.16) | 156 / 553 (28.21) |
| 21 | 74 / 129 (57.36) | 930 / 1428 (65.13) | 148 / 701 (21.11) |
| 22 | 98 / 120 (81.67) | 1102 / 1397 (78.88) | 196 / 513 (38.21) |
| 23 | 32 / 32 (100.00) | 3051 / 3823 (79.81) | 64 / 836 (7.66) |
| 24 | 57 / 74 (77.03) | 808 / 1038 (77.84) | 114 / 361 (31.58) |
| 25 | 66 / 79 (83.54) | 1063 / 1330 (79.92) | 132 / 412 (32.04) |
| 26 | 69 / 87 (79.31) | 1320 / 1516 (87.07) | 138 / 352 (39.20) |
| 27 | 85 / 100 (85.00) | 1252 / 1751 (71.50) | 170 / 684 (24.85) |
| 28 | 232 / 244 (95.08) | 2469 / 3186 (77.50) | 464 / 1193 (38.89) |
| 29 | 55 / 78 (70.51) | 893 / 1218 (73.32) | 110 / 458 (24.02) |
| 30 | 206 / 259 (79.54) | 2318 / 3005 (77.12) | 412 / 1152 (35.76) |
| 31 | 62 / 91 (68.13) | 830 / 1298 (63.94) | 124 / 621 (19.97) |
| 32 | 506 / 651 (77.73) | 1898 / 2434 (77.98) | 1012 / 1693 (59.78) |
| 33 | 184 / 230 (80.00) | 2141 / 2891 (74.06) | 368 / 1164 (31.62) |
| 34 | 419 / 467 (89.72) | 2109 / 2638 (79.95) | 838 / 1415 (59.22) |
| 35 | 233 / 259 (89.96) | 1342 / 2995 (44.81) | 466 / 2145 (21.72) |
| 36 | 297 / 401 (74.06) | 2596 / 3282 (79.10) | 594 / 1384 (42.92) |
| 37 | 317 / 407 (77.89) | 2116 / 2763 (76.58) | 634 / 1371 (46.24) |
| 38 | 169 / 226 (74.78) | 2059 / 2970 (69.33) | 338 / 1306 (25.88) |
| 39 | 307 / 403 (76.18) | 2433 / 3607 (67.45) | 614 / 1884 (32.59) |
| 40 | 170 / 180 (94.44) | 1872 / 2515 (74.43) | 340 / 993 (34.24) |

**Supplementary table 2B. Sensitivity, and specificity for hand motor area identification in the ipsilesional hemisphere.**

Note. – Values are reported as numerator/denominator (%). Sensitivity = TP / (TP + FN); Specificity = TN / (TN + FP); and Dice coefficient = 2 × A∩B / (A + B). ID = subject identifier; TP = true positives (A∩B); TN = true negatives; FP = false positives; FN = false negatives. Corresponding values are shown in supplementary table 2A.

**Supplementary table 3A. Confusion matrix values for hand motor area detection in the contralesional hemisphere.**

| ID | A | B | TP | TN | FP | FN |
| --- | --- | --- | --- | --- | --- | --- |
| 1 | 131 | 377 | 105 | 709 | 272 | 26 |
| 2 | 287 | 388 | 220 | 665 | 168 | 67 |
| 3 | 114 | 288 | 67 | 823 | 221 | 47 |
| 4 | 177 | 355 | 114 | 969 | 241 | 63 |
| 5 | 178 | 350 | 122 | 829 | 228 | 56 |
| 6 | 80 | 369 | 69 | 750 | 300 | 11 |
| 7 | 180 | 440 | 136 | 886 | 304 | 44 |
| 8 | 178 | 304 | 140 | 965 | 164 | 38 |
| 9 | 167 | 396 | 89 | 797 | 307 | 78 |
| 10 | 181 | 402 | 131 | 1087 | 271 | 50 |
| 11 | 170 | 327 | 147 | 813 | 180 | 23 |
| 12 | 119 | 366 | 98 | 697 | 268 | 21 |
| 13 | 51 | 396 | 47 | 692 | 349 | 4 |
| 14 | 238 | 410 | 143 | 679 | 267 | 95 |
| 15 | 174 | 382 | 87 | 939 | 295 | 87 |
| 16 | 101 | 500 | 56 | 1134 | 444 | 45 |
| 17 | 119 | 371 | 66 | 868 | 305 | 53 |
| 18 | 98 | 318 | 52 | 1087 | 266 | 46 |
| 19 | 82 | 417 | 56 | 1210 | 361 | 26 |
| 20 | 117 | 378 | 85 | 1377 | 293 | 32 |
| 21 | 233 | 526 | 181 | 853 | 345 | 52 |
| 22 | 165 | 420 | 90 | 1141 | 330 | 75 |
| 23 | 129 | 664 | 80 | 302 | 584 | 49 |
| 24 | 139 | 260 | 101 | 1050 | 159 | 38 |
| 25 | 87 | 243 | 44 | 1239 | 199 | 43 |
| 26 | 79 | 159 | 48 | 1400 | 111 | 31 |
| 27 | 85 | 571 | 81 | 959 | 490 | 4 |
| 28 | 336 | 1024 | 271 | 2661 | 753 | 65 |
| 29 | 56 | 325 | 44 | 1046 | 281 | 12 |
| 30 | 199 | 924 | 180 | 1959 | 744 | 19 |
| 31 | 133 | 386 | 104 | 1176 | 282 | 29 |
| 32 | 374 | 1101 | 339 | 1601 | 762 | 35 |
| 33 | 277 | 899 | 233 | 2417 | 666 | 44 |
| 34 | 327 | 982 | 310 | 2372 | 672 | 17 |
| 35 | 194 | 1436 | 193 | 2007 | 1243 | 1 |
| 36 | 814 | 875 | 534 | 1721 | 341 | 280 |
| 37 | 299 | 1178 | 268 | 2180 | 910 | 31 |
| 38 | 222 | 833 | 159 | 1750 | 674 | 63 |
| 39 | 273 | 1077 | 257 | 2161 | 820 | 16 |
| 40 | 379 | 919 | 237 | 1989 | 682 | 142 |
|  |  |  |  |  |  |  |

Note. – Columns 2 to 7 data are number of voxels. Values used to compute contralesional sensitivity, and specificity in supplementary table 3B. ID = subject identifier, A = hand motor task voxels, B = scICA voxels of the predicted SMN, TP = true positive (A∩B), TN = true negative, FP = false positive, FN = false negative.

**Supplementary table 3B. Sensitivity, and specificity for hand motor area identification in the contralesional hemisphere.**

| ID | Sensitivity | Specificity | Dice coefficient |
| --- | --- | --- | --- |
| 1 | 105 / 131 (80.15) | 709 / 981 (72.27) | 210 / 508 (41.34) |
| 2 | 220 / 287 (76.66) | 665 / 833 (79.83) | 440 / 675 (65.19) |
| 3 | 67 / 114 (58.77) | 823 / 1044 (78.83) | 134 / 402 (33.33) |
| 4 | 114 / 177 (64.41) | 969 / 1210 (80.08) | 228 / 532 (42.86) |
| 5 | 122 / 178 (68.54) | 829 / 1057 (78.43) | 244 / 528 (46.21) |
| 6 | 69 / 80 (86.25) | 750 / 1050 (71.43) | 138 / 449 (30.73) |
| 7 | 136 / 180 (75.56) | 886 / 1190 (74.45) | 272 / 620 (43.87) |
| 8 | 140 / 178 (78.65) | 965 / 1129 (85.47) | 280 / 482 (58.09) |
| 9 | 89 / 167 (53.29) | 797 / 1104 (72.19) | 178 / 563 (31.62) |
| 10 | 131 / 181 (72.38) | 1087 / 1358 (80.04) | 262 / 583 (44.94) |
| 11 | 147 / 170 (86.47) | 813 / 993 (81.87) | 294 / 497 (59.15) |
| 12 | 98 / 119 (82.35) | 697 / 965 (72.23) | 196 / 485 (40.41) |
| 13 | 47 / 51 (92.16) | 692 / 1041 (66.47) | 94 / 447 (21.03) |
| 14 | 143 / 238 (60.08) | 679 / 946 (71.78) | 286 / 648 (44.14) |
| 15 | 87 / 174 (50.00) | 939 / 1234 (76.09) | 174 / 556 (31.29) |
| 16 | 56 / 101 (55.45) | 1134 / 1578 (71.86) | 112 / 601 (18.64) |
| 17 | 66 / 119 (55.46) | 868 / 1173 (74.00) | 132 / 490 (26.94) |
| 18 | 52 / 98 (53.06) | 1087 / 1353 (80.34) | 104 / 416 (25.00) |
| 19 | 56 / 82 (68.29) | 1210 / 1571 (77.02) | 112 / 499 (22.44) |
| 20 | 85 / 117 (72.65) | 1377 / 1670 (82.46) | 170 / 495 (34.34) |
| 21 | 181 / 233 (77.68) | 853 / 1198 (71.20) | 362 / 759 (47.69) |
| 22 | 90 / 165 (54.55) | 1141 / 1471 (77.57) | 180 / 585 (30.77) |
| 23 | 80 / 129 (62.02) | 3302 / 3886 (84.97) | 160 / 793 (20.18) |
| 24 | 101 / 139 (72.66) | 1050 / 1209 (86.85) | 202 / 399 (50.63) |
| 25 | 44 / 87 (50.57) | 1239 / 1438 (86.16) | 88 / 330 (26.67) |
| 26 | 48 / 79 (60.76) | 1400 / 1511 (92.65) | 96 / 238 (40.34) |
| 27 | 81 / 85 (95.29) | 959 / 1449 (66.18) | 162 / 656 (24.70) |
| 28 | 271 / 336 (80.65) | 2661 / 3414 (77.94) | 542 / 1360 (39.85) |
| 29 | 44 / 56 (78.57) | 1046 / 1327 (78.82) | 88 / 381 (23.10) |
| 30 | 180 / 199 (90.45) | 1959 / 2703 (72.48) | 360 / 1123 (32.06) |
| 31 | 104 / 133 (78.20) | 1176 / 1458 (80.66) | 208 / 519 (40.08) |
| 32 | 339 / 374 (90.64) | 1601 / 2363 (67.75) | 678 / 1475 (45.97) |
| 33 | 233 / 277 (84.12) | 2417 / 3083 (78.40) | 466 / 1176 (39.63) |
| 34 | 310 / 327 (94.80) | 2372 / 3044 (77.92) | 620 / 1309 (47.36) |
| 35 | 193 / 194 (99.48) | 2007 / 3250 (61.75) | 386 / 1630 (23.68) |
| 36 | 534 / 814 (65.60) | 1721 / 2062 (83.46) | 1068 / 1689 (63.23) |
| 37 | 268 / 299 (89.63) | 2180 / 3090 (70.55) | 536 / 1477 (36.29) |
| 38 | 159 / 222 (71.62) | 1750 / 2424 (72.19) | 318 / 1055 (30.14) |
| 39 | 257 / 273 (94.14) | 2161 / 2981 (72.49) | 514 / 1350 (38.07) |
| 40 | 237 / 379 (62.53) | 1989 / 2671 (74.47) | 474 / 1298 (36.52) |
|  |  |  |  |

Note. – Values are reported as numerator/denominator (%). Sensitivity = TP / (TP + FN); Specificity = TN / (TN + FP); and Dice coefficient = 2 × A∩B / (A + B). ID = subject identifier; TP = true positives (A∩B); TN = true negatives; FP = false positives; FN = false negatives. Corresponding values are shown in supplementary table 3A.

**Media Supplementary material.**

A videoclip of the 3D overlap images of patients 11, 23, 28 and 34. This is an interactive clip of the 3D rendered brains in Figure 3 of the manuscript. Only ipsilesional hemisphere is shown.
